# Granulomatosis with polyangiitis presenting as an orbital inflammatory pseudotumor: a case report

**DOI:** 10.1186/1752-1947-7-110

**Published:** 2013-04-23

**Authors:** Naohi Isse, Yuichi Nagamatsu, Naoko Yoshimatsu, Toshiyuki Obata, Noriko Takahara

**Affiliations:** 1 Department of Internal Medicine, Ako City Hospital, 1090 Nakahiro, Ako, Hyogo, 6780-232, Japan

## Abstract

**Introduction:**

Granulomatosis with polyangiitis is a systemic inflammatory disease that often presents with necrosis, granuloma formation and vasculitis of small- to medium-sized vessels. Affected patients usually present with disease of the upper respiratory tract, lungs and kidneys, but this disease has been reported to involve almost any organ. We report the case of a patient with ocular manifestations of granulomatosis with polyangiitis after the remission of renal and auditory manifestations.

**Case presentation:**

An 81-year-old Japanese woman had a four-year history of biopsy-proven antineutrophil cytoplasmic antibody-related glomerulonephritis that had been treated with oral prednisolone and was in serological remission. She had also recovered from a one-year history of complete hearing loss immediately following the steroid treatment for glomerulonephritis. She gradually experienced right eye visual disturbance and exophthalmos over a two-month period. Radiographic and histopathological findings revealed an orbital inflammatory pseudotumor. The administration of prednisolone completely restored her right eye visual acuity and eye movement after two weeks. Considering this case retrospectively, our patient had an orbital inflammatory pseudotumor caused by granulomatosis with polyangiitis including a medical history of reversible hearing loss, although her glomerulonephritis had remitted with an undetectable level of specific antineutrophil cytoplasmic antibody.

**Conclusions:**

In this patient, hearing loss and visual loss occurred at different times during the course of treatment of granulomatosis with polyangiitis. Clinicians should consider a differential diagnosis of granulomatosis with polyangiitis in patients with treatable hearing and visual loss.

## Introduction

Granulomatosis with polyangiitis (GPA), formerly called Wegener’s granulomatosis, was first described by Heinz Klinger in 1932. GPA usually presents as a triad of airway necrotizing granulomas, systemic vasculitis of small- to medium-sized vessels, and glomerulonephritis. Classic symptoms, clinical findings and serology titers positive for antineutrophil cytoplasmic antibody (ANCA) against proteinase 3 (PR3-ANCA) confirm the diagnosis. Affected patients usually present with disease of the upper respiratory tract, lungs and kidneys, but GPA has been reported to involve almost any organ [1]. Some reports have shown GPA occurrence in patients with hearing loss [2], and others have shown its occurrence in the orbital space [3]. Our patient presented with an unusual clinical course: complete hearing loss followed by glomerulonephritis accompanied by an elevated serum titer of myeloperoxidase (MPO)-ANCA and subsequent visual loss with an undetectable level of MPO-ANCA.

## Case presentation

An 81-year-old Japanese woman had a four-year history of biopsy-proven ANCA-related glomerulonephritis with no lung lesions. She had initially been hospitalized to investigate a recent onset of microscopic hematuria, proteinuria (0.8g per day) and an increase in serum creatinine from 0.5 to 1.1mg/dL in one month. Renal biopsy specimens revealed pauci-immune crescentic glomerulonephritis with segmental glomerular necrosis and a medium-sized artery obliterated by concentric inflammation. Her condition was treated with oral prednisolone, which resulted in serological remission. Before the steroid treatment, her MPO-ANCA titer had been significantly elevated at 140 enzyme-linked immunosorbent assay units (EU; normal, 0 to 19 EU), but it had improved to an undetectable level over the previous year with 7mg of oral prednisolone. Neither proteinuria nor hematuria was observed during the previous year. She had also recovered from a one-year history of complete hearing loss during the initial high-dose prednisolone treatment for ANCA-related glomerulonephritis.

**Figure 1:**
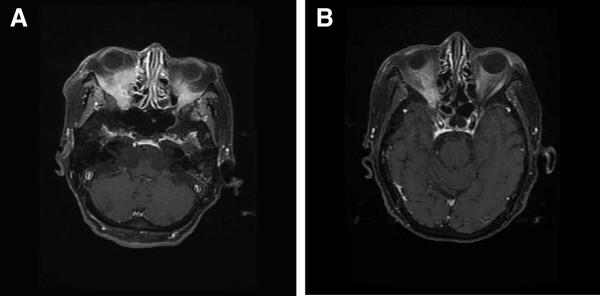
**T1**-**weighted magnetic resonance images of the orbit with gadolinium infusion and fat suppression.** (**A**) Coronal image taken at initial presentation showing a mass involving the right orbital space and extending into the ethmoidal sinus. The mass is displacing the globe. A small mass is also shown in the left orbital space. (**B**) Coronal image showing reduction of the pseudotumor 12 weeks after treatment.

On the current admission, our patient presented with a two-month history of gradually progressive right eye visual disturbance and exophthalmos. Magnetic resonance (MR) images revealed a right orbital tumor that invaded the ethmoidal sinus (Figure 1A). Our patient had undergone cataract surgery of her right eye six months previously, so she was concerned about developing a bacterial infection of her eyelid. She was treated with a seven-day course of oral sultamicillin tosilate hydrate; however, it was ineffective against her ophthalmological symptoms. Her other medical history included a right hip fracture two years previously and well-controlled type 2 diabetes mellitus.

She was afebrile and without weight loss, but felt lethargic while walking around. Her right eye visual acuity was limited to hand movements and had markedly deteriorated over two months. Her right eye lateral gaze movement was also restricted. Right eye proptosis was also observed, but there were no ocular lesions such as conjunctivitis or episcleritis. An examination of her neck, ears and nose by an ear, nose and throat surgeon using flexible nasal endoscopy was normal. She did not have saddle nose. A full blood count showed normal values; her plasma C-reactive protein level was slightly increased at 5.0mg/dL (Table 1). A chest X-ray showed neither infiltration nor nodules in her lungs. Her renal function and urine findings were also normal.

**Table 1:** Blood and urine laboratory findings at the time of initial presentation

White blood cells	9200	/μL	Blood glucose	98	mg/dL
Red bood cells	410×10^4^	/μL	Hemoglobin A1c^a^	6.8	%
Hemoglobin	12.3	g/dL	Thyroid stimulating hormone	0.32	μU/mL
Platelet	27.2×10^4^	/μL	Free thyroxine	1.11	ng/dL
Total protein	6.3	g/dL	MPO-ANCA	<10	EU
Albumin	3.3	g/dL	PR3-ANCA	<10	EU
Creatinine kinase	25	U/L	Immunoglobulin A	290	mg/dL
Total bilirubin	0.9	mg/dL	Immunoglobulin M	47	mg/dL
Aspartate aminotransferase	15	U/L	Immunoglobulin G	1245	mg/dL
Alanine aminotransferase	10	U/L	Immunoglobulin G4	13.3	mg/dL
Lactate dehydrogenase	177	U/L			
γ-glutamyl transpeptidase	20	U/L	Urine protein	(−)	
C-reactive protein	5.0	mg/dL	Urine blood	(−)	
Sodium ion	138	mEq/L			
Potassium ion	3.3	mEq/L			
Chloride ion	99	mEq/L			
Blood urea nitrogen	22.6	mg/dL			
Serum creatinine	0.68	mg/dL			

^a^National Glycohemoglobin Standardization Program. MPO-ANCA: myeloperoxidase antineutrophil cytoplasmic antibody; PR3-ANCA: antineutrophil cytoplasmic antibody against proteinase 3.

**Figure 2:**
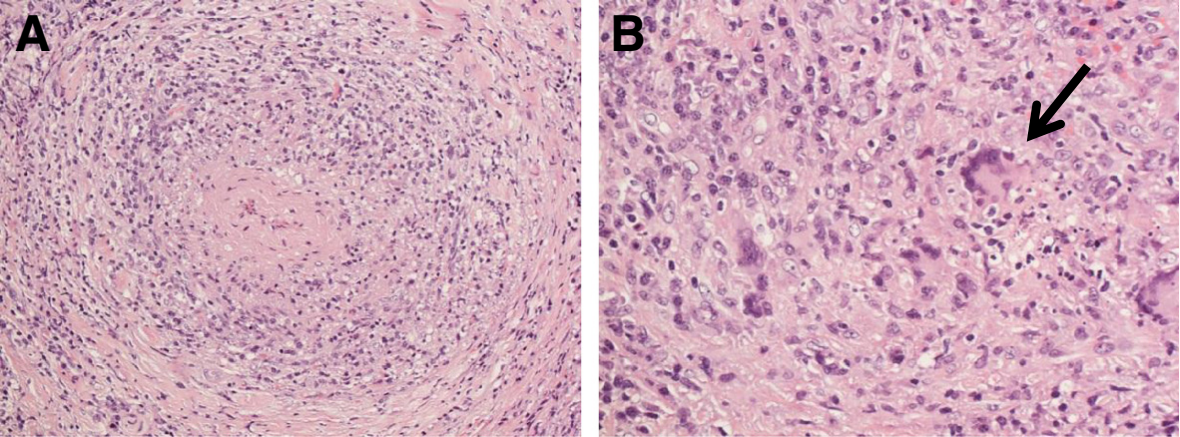
**Histology of the right orbital tumor.** Photomicrographs of the right orbital pseudotumor showing vasculitis of a medium-sized artery (hematoxylin and eosin staining). (**A**) The medium-sized artery is obliterated by concentric inflammation. (**B**) Granulomatous inflammation with a multinucleated giant cell is noted (arrow).

**Figure 3:**
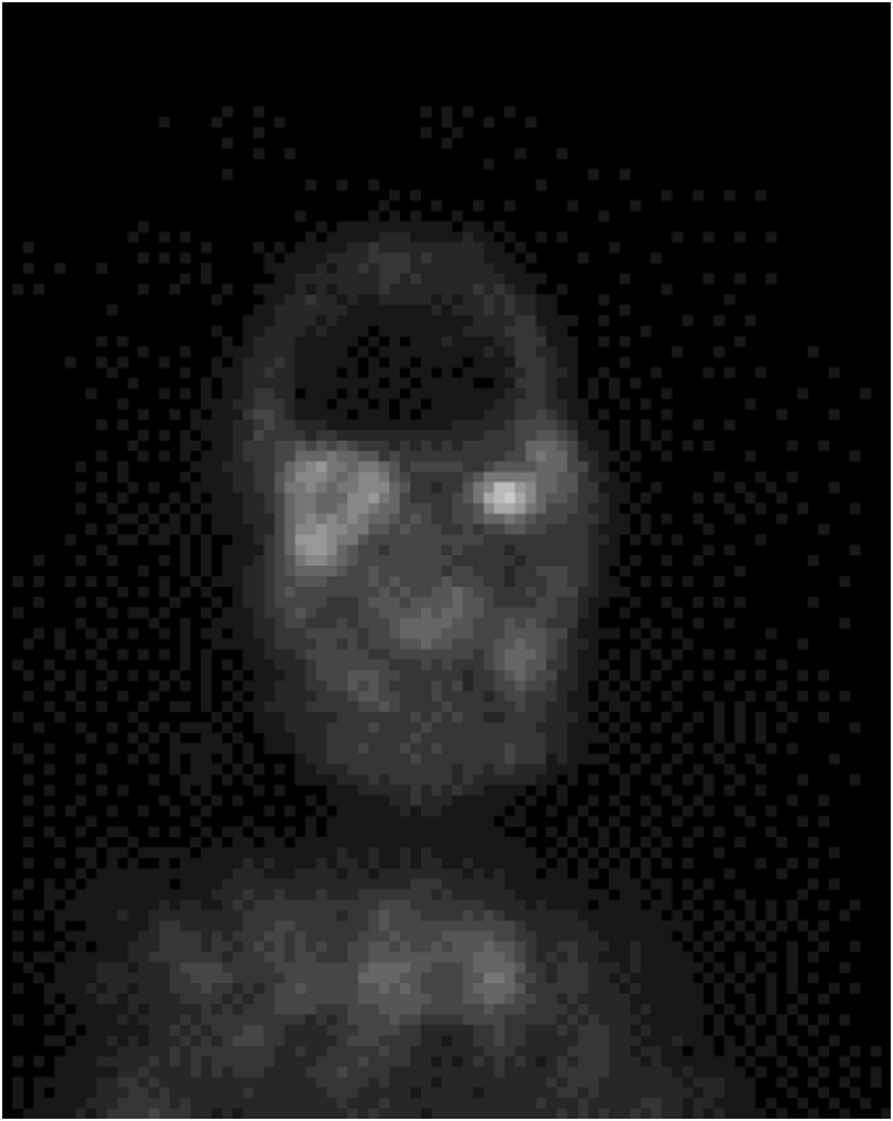
**Radioisotope inflammatory scintigraphy image.** Intense uptake of gallium-67 in the bilateral but right side-dominant orbital lesion.

The differential diagnoses included a malignant orbital tumor, immunoglobulin G4-related autoimmune disease and an inflammatory pseudotumor. To conduct a histopathological examination, a tumor biopsy through an orbitotomy was performed by a plastic surgeon under general anesthesia. The histopathology revealed severe inflammation around the small vessels, which was compatible with systemic vasculitis (Figure 2). Immunohistochemical staining ruled out malignant lymphoma and immunoglobulin G4-related autoimmune disease. Gallium-67 inflammatory scintigraphy findings revealed a bilateral but right side-dominant orbital inflammatory lesion (Figure 3). These findings were compatible with the diagnosis of a pseudoinflammatory tumor related to systemic vasculitis. A three-day course of 500mg of intravenous methylprednisolone was initiated followed by 30mg of oral prednisolone, which completely restored her right eye visual acuity and eye movement after two weeks. The administration of oral prednisolone was tapered to 10mg within eight weeks. Follow-up MR images demonstrated significant reduction of the orbital pseudotumor (Figure 1B).

Considering this case retrospectively, our patient had GPA rather than microscopic polyangiitis; the GPA also accounted for her medical history of reversible hearing loss, although her glomerulonephritis remitted with an undetectable level of MPO-ANCA. These lesions are consistent with a diagnosis of GPA by the American College of Rheumatology criteria. She was successfully discharged home.

## Discussion

The often rapidly progressive and potentially fatal disease GPA affects mainly the upper and lower respiratory tracts and kidneys. Early recognition and treatment is important to prevent severe organ damage. Head and neck symptoms occur in 90% of patients; the nose, paranasal sinuses (up to 80% of patients) and middle ear are commonly affected [1,4]. In a previous report, GPA occurred in the form of both conductive and sensorineural hearing loss without systemic features [2]. Involvement of ocular and orbital structures is common; it is estimated to occur in 29% to 52% of patients with GPA and may be a presenting feature [5,6]. Immunoglobulin G4-related systemic disease has also been known to cause a similar type of orbital pseudotumor on MR images, which can be distinguished from GPA using histopathology or serology [7]. In our patient, GPA initially presented with bilateral hearing loss. Without investigation or treatment, it progressed to glomerulonephritis after one year. After remission by steroid treatment, our patient relapsed and presented with an orbital pseudotumor without re-elevation of MPO-ANCA. The PR3-ANCA titer, which is usually relatively elevated in GPA, was also negative at this time. It is interesting to note that the biopsy specimens of both the kidney and the orbital pseudotumor showed similar active inflammation around a medium-sized artery despite the four-year span between each presentation. Each episode could be considered as limited GPA. An increase in her C-reactive protein level was the only serological marker of GPA relapse. Neither lung nor sinonasal lesions developed during the disease course.

Generally, it is believed that the treatment of choice for significant ocular GPA is a combination of glucocorticoids and cyclophosphamide [5]. However, among patients with limited Wegener’s granulomatosis, remission is induced in approximately three quarters of patients using methotrexate and glucocorticoids alone [1]. Considering susceptibility to infectious diseases due to our patient’s age, long-term steroid use concerns, diabetes mellitus and poor daily activity, we decided to begin with steroid monotherapy. Steroid therapy alone was markedly effective in our patient. Her presentation and quick response to steroid therapy alone are rare [1,8].

In elderly patients, the use of glucocorticoids alone may be preferable, considering the various potential systemic side effects of cyclophosphamide, such as pneumonia and urinary tract infection due to neutropenia. Thus, based on this case, we suggest alternative treatments for ophthalmic complications in GPA. In such cases, careful follow-up is needed to identify any forms of relapse, including that involving other organs. As shown in the rare presentation described in our case report, the MPO-ANCA titer may not be a marker of relapse.

## Conclusion

Our elderly patient’s clinical course was uncommon but it is clinically important in that hearing and visual loss occurring at different times were successfully treated by steroid therapy. Early recognition and treatment is particularly important because GPA progresses to involve other organs as a fatal disease. Clinicians should consider GPA as a differential diagnosis in patients with treatable hearing and visual loss.

### Patient’s perspective

Five years ago, in the autumn, I began to gradually lose my hearing. After one month, I could hear no sounds at all and I was very worried about my future. I became discouraged because I was unable to enjoy listening to my favorite music or write letters and I felt sad that I could not interact with my family. I felt weakness in my legs and went to the hospital because I could not walk by myself. I was told that I had diabetes mellitus. After treatment, my right ear gradually recovered hearing and I could talk with my family again.

In the spring, I felt such severe dizziness that I could not even sit on a chair. I lost my appetite, I was bothered by eye discharge and my dizziness was not relieved even when I lay down. I experienced difficulty writing calligraphy because of weakness in my hands. I did not feel like doing anything.

Since the operation, I am now able to read the newspaper and enjoy watching television and I am very happy and grateful to my doctor, nurses and physical therapists. Thank you very much.

## Consent

Written informed consent was obtained from the patient for publication of this case report and accompanying images. A copy of the written consent is available for review by the Editor-in-Chief of this journal.

## Abbreviations

ANCA: Antineutrophil cytoplasmic antibody; GPA: Granulomatosis with polyangiitis; MPO-ANCA: Myeloperoxidase antineutrophil cytoplasmic antibody; MR: Magnetic resonance; PR3-ANCA: Antineutrophil cytoplasmic antibody against proteinase 3.

## Competing interests

The authors declare that they have no competing interests.

## Authors’ contributions

NI was a major contributor to the writing of the manuscript. YN was primarily involved in the care of the patient. NY and TO were involved in data interpretation. NT was involved in administrative support. All authors read and approved the final manuscript.
